# Minor Groove Binder Distamycin Remodels Chromatin but Inhibits Transcription

**DOI:** 10.1371/journal.pone.0057693

**Published:** 2013-02-27

**Authors:** Parijat Majumder, Amrita Banerjee, Jayasha Shandilya, Parijat Senapati, Snehajyoti Chatterjee, Tapas K. Kundu, Dipak Dasgupta

**Affiliations:** 1 Biophysics Division, Saha Institute of Nuclear Physics, Kolkata, West Bengal, India; 2 Transcription and Disease Laboratory, Molecular Biology and Genetics Unit, Jawaharlal Nehru Centre for Advanced Scientific Research, Bangalore, Karnataka, India; Bioinformatics Institute, Singapore

## Abstract

The condensed structure of chromatin limits access of cellular machinery towards template DNA. This in turn represses essential processes like transcription, replication, repair and recombination. The repression is alleviated by a variety of energy dependent processes, collectively known as “chromatin remodeling”. In a eukaryotic cell, a fine balance between condensed and de-condensed states of chromatin helps to maintain an optimum level of gene expression. DNA binding small molecules have the potential to perturb such equilibrium. We present herein the study of an oligopeptide antibiotic distamycin, which binds to the minor groove of B-DNA. Chromatin mobility assays and circular dichroism spectroscopy have been employed to study the effect of distamycin on chromatosomes, isolated from the liver of Sprague-Dawley rats. Our results show that distamycin is capable of remodeling both chromatosomes and reconstituted nucleosomes, and the remodeling takes place in an ATP-independent manner. Binding of distamycin to the linker and nucleosomal DNA culminates in eviction of the linker histone and the formation of a population of off-centered nucleosomes. This hints at a possible corkscrew type motion of the DNA with respect to the histone octamer. Our results indicate that distamycin in spite of remodeling chromatin, inhibits transcription from both DNA and chromatin templates. Therefore, the DNA that is made accessible due to remodeling is either structurally incompetent for transcription, or bound distamycin poses a roadblock for the transcription machinery to advance.

## Introduction

Hierarchical packaging of DNA in the form of chromatin enables the cell nucleus to accommodate nearly 2 m of DNA [1]. The lowest level of packing occurs in the nucleosome, where a short stretch of DNA (∼146 bp) is wrapped in 1.65 turns of a left handed superhelix around an octameric core of histone proteins [2]. Repetition of this local packing motif, along with a stretch of linker DNA, gives rise to higher order folded structures [3], [4]. The primary unit of higher order chromatin is the chromatosome, where a linker histone interacts asymmetrically with a nucleosome [5]. In condensed chromatin fibers, presence of H1 ensures that the DNA entry and exit sites are in proximity, thereby posing constraints on the spatial orientation of nucleosomes. In a chromatosome, the linker histone seals the DNA that wraps around the histone octamer, preventing its transient dissociation [6], [7]. This topological ordering renders the DNA partially inaccessible to DNA-binding proteins, and in turn, hampers the process of gene expression [1], [8].

Several cellular mechanisms increase the accessibility of nucleosomal DNA to protein factors [9], [10], [11]. They are (i) chromatin breathing, which refers to the transient dissociation and re-association of the ends of nucleosomal DNA; (ii) nucleosomal remodeling, which may be spontaneous or catalyzed and (iii) changes in the higher order structure of chromatin.

Nucleosomal remodeling is caused by a set of specialized chromatin remodeling complexes that translocate, destabilize, dissociate or restructure nucleosomes [12], [13]. These factors may be targeted to specific loci to remodel a single or very few nucleosomes at strategic sites. Others may perform untargeted remodeling throughout large chromosomal domains. However, the common feature of all chromatin remodeling complexes is their ability to hydrolyze ATP and utilize the energy generated therein to alter histone-DNA contacts.

Chromatin fluidity and proper nucleosomal positioning are critical to the fidelity of eukaryotic transcription [14], [15]. Transcription factors initially recognize and bind to DNA promoters that are characteristically nucleosome free regions [16]. Transcription elongation requires a mechanism for the advancing polymerase complex to overcome the nucleosome barrier. In case of the bacteriophage T7 RNA polymerase [17], [18], [19], and the eukaryotic RNA polymerase III [20], the elongation complex initially disrupts histone-DNA contacts about 20 bp ahead of the polymerase. As the complex reaches the nucleosome dyad, the histone octamer is displaced in *cis* to a DNA region behind the RNA polymerase, giving rise to an intermediate loop. The loop region is subsequently transcribed. In case of RNA polymerase II however, the presence of nucleosomes block transcription at physiological ionic strength [21], [22]. The barrier is overcome at higher ionic strength, but transcription through nucleosomal template results in eviction of H2A-H2B dimer [23]. Although the mechanism of transcription through chromatin template is not clearly understood, yet the indispensible involvement of remodeling is well accepted.

In our laboratory, we have been studying the effects of DNA binding small molecules upon chromatin structure at different levels [24], [25], [26], [27]. Here, we ask whether there exists any functional relationship between nucleosomal DNA accessibility and chromatin transcription when these molecules bind to DNA. We have chosen the oligopeptide antibiotic, distamycin A, which inhibits the pathogenesis of vaccinia virus in culture [28]. It displaces essential transcription factors like SRF and MEF2 [29], and inhibits the binding of high mobility group proteins HMGA1 to P-Selectin promoter [30]. It also inhibits the binding of DNA to nuclear scaffold and linker histones [31]. Distamycin binds isohelically to the minor groove of DNA, preferably at A/T rich regions [32], [33], [34], [35], [36], [37]. Its binding to DNA widens the minor groove and bends back the helix axis [32]. The helix axis is lengthened by nearly 12–15% [38]. In the context of gene expression, distamycin is known to inhibit transcription initiation from DNA template, but not elongation [39], [40]. It inhibits TBP binding and basal *in vitro* transcription [41]. Although the effect of this molecule has been well studied at the DNA level [42], [43], [44], [45], [46], [47], [48], the interaction at the chromatin level is still obscure. Recently we have shown that distamycin binds to chromatin and chromosomal DNA with comparable affinity, implying that the site for drug binding is equally accessible in both cases [26]. Previous studies with nucleosomes, reconstituted on tyrT DNA have revealed that distamycin alters the rotational positioning of nucleosomal DNA with respect to the octamer surface [49], [50].

In the present report, we have shown that distamycin A, remodels chromatosomes and mononucleosomes causing the histone octamer to translocate on the DNA in an ATP-independent manner. However, distamycin binding inhibits transcription through DNA and chromatin templates. Our results imply that in the context of small molecules, enhancement of DNA accessibility may be a prerequisite, but is not sufficient for transcription to take place.

## Materials and Methods

### Chromatosome Preparation

Chromatosome was isolated from liver tissue of male albino Sprague-Dawley rats, obtained from the animal house facility of the Indian Institute of Chemical Biology, Kolkata. Sprague-Dawley rats, weighing 125–150 grams were maintained in a conducive environment (*i.e*. 24±2°C temperature; 55–60% relative humidity; and 12∶12 hrs light and dark schedule) and were provided *ad libitum* with balanced and sterilized diet, produced in-house. All rats were acclimatized in such conditions for at least one week prior to dissection. For isolation of liver, the rats were sacrificed by cervical dislocation and the livers were stored in sealed tubes at −80°C. Please note that for all experiments using rat, internationally recognized guidelines were followed. The experiments were performed with the approval for ethical clearance from Institutional Animal Ethics Committee (IAEC), Jawaharlal Nehru Centre for Advanced Scientific Research, Bangalore, India (Reference number: IAEC/2011/TKK/002).

Rat liver nuclei were digested with micrococcal nuclease, and purified by centrifugation through a 5–30% sucrose density gradient, prepared in buffer (5 mM Tris HCl (pH 7.4), 15 mM NaCl and 1 mM EDTA) [26]. Prior to experiments, the samples were dialysed against the same buffer, and the mononucleotide concentration was determined spectrophotometrically, using the molar extinction coefficient of ε_260_ = 6600 M^−1^ cm^−1^.

### Preparation of Histones

Histone octamers were prepared from chicken erythrocytes by standard methods [51] and dialysed against 10 mM Tris HCl (pH 7.4) containing 2 M NaCl. The concentration was determined using the extinction coefficient of ε_230_ = 507553 M^−1^ cm^−1^. Linker histone H1 was purchased from New England Biolabs. It was dialysed in 5 mM Tris HCl (pH 7.4), 100 mM NaCl, and the concentration was determined spectrophotometrically using the extinction coefficient of ε_280_ = 3840 M^−1^ cm^−1^.

### Mononucleosome Reconstitution

A 200 bp DNA fragment, containing the 601 positioning sequence in the center, was constructed by PCR, and the amplification product was purified by PCR purification kit (Qiagen). The concentration of DNA constructs was determined using molar extinction coefficients of ε_260_ = 3188500 M^−1^ cm^−1^, obtained by neighbor approximation method.

Nucleosomes were assembled by salt dialysis method [52]. The DNA and histone octamers were mixed in a molar ratio of 1∶1.2 and incubated with equal volume of 2X initial assembly buffer (20 mM Tris HCl (pH 7.4), 2 mM EDTA (pH 8.0), 4 M NaCl, 20 mM β-mercaptoethanol, and 2 mg/ml BSA) for 30 minutes at 37°C. The initial assembly reaction was followed by step dialysis against 10 mM Tris HCl (pH 7.4), 1 mM EDTA (pH 8.0), 10 mM β-mercaptoethanol, 0.5 mM PMSF containing decreasing concentrations of NaCl (1.8 M, 1.4 M, 1.0 M, 0.8 M, 0.6 M, 0.3 M, and 0 M). Reconstituted nucleosomes were purified by centrifugation through a 5–30% sucrose density gradient in 5 mM Tris HCl (pH 7.4), 15 mM NaCl, 1 mM EDTA and finally dialysed against 5 mM Tris HCl (pH 7.4), 15 mM NaCl, 1 mM EDTA.

### Chromatin mobility Assay

A solution of distamycin A (Sigma) was prepared in 20 mM NaCl containing 5 mM Tris HCl (pH 7.4), and the concentration was determined using molar extinction coefficient of 34000 M^−1^ cm^−1^ at 303 nm [33]. Chromatosomes (300 µM base), isolated from rat liver, were incubated with distamycin in drug to DNA base ratio of 0, 0.08, 0.16 and 0.25 (0 µM, 25 µM, 50 µM and 75 µM respectively) for 90 minutes at room temperature. The samples were then analyzed by electrophoresis on 1.5% agarose gel in 0.5X TBE, followed by staining with SYBR green. A control experiment was performed with chromatosomal DNA.

To observe the dynamics of the conformational change, a time-course experiment was performed, whereby, chromatosome (300 µM base) was incubated with 50 µM distamycin for varying time periods and the reaction mixtures were electrophoresed on 1.5% agarose gel.

To determine the condition of DNA and protein in the resultant populations of the chromatosome mobility assay, the bands were excised and electroeluted in 1X TBE. The electroeluted samples were then extracted with phenol-chloroform-isoamyl alcohol to isolate the DNA component. Similarly, the protein component was isolated by TCA precipitation of electroeluted samples. The DNA and protein components were separately analyzed by electrophoresis on 1.5% agarose gel and 18% SDS-PAGE respectively. Histone composition was further confirmed by western blot analysis using anti-histone antibodies H1 [(C-17): sc-8616], H2A [(N-15): sc-8647], H2B [(N-20): sc-8650], H3 [(N-20): sc-8653] and H4 [(N-18): sc-8657] (dilution 1∶200 in 3% skim milk prepared in TBST). Signals were generated using chemiluminescent substrates from Thermo Scientific (SuperSignal West Pico Substrate) in a dark room on X-ray films providing short exposures of 10 seconds and the blots were developed using developer and fixer solutions from Millipore.

To study the ATP dependence of the destabilization process, chromatosomes were incubated with 2 units/ml apyrase for 30 minutes at 30°C [53]. The apyrase treated chromatosomes (300 µM DNA base) were then incubated with distamycin (0, 25 µM, 50 µM and 75 µM) to achieve a drug to DNA base ratio of 0, 0.08, 0.16 and 0.25 respectively. The incubation was done for 90 minutes at room temperature and the reaction mixtures were then electrophoresed on 1.5% agarose gel.

Furthermore, mononucleosomes, reconstituted on 200 bp 601 DNA fragment were also treated with distamycin in similar proportions and for similar time periods and electrophoresed on 1.5% agarose gel.

### Isothermal Titration Calorimetry

Histone octamer and linker histone were individually dialysed against 5 mM Tris HCl (pH 7.4), 100 mM NaCl. To study histone- distamycin interaction, ITC experiments were performed in an ITC200 from MicroCal, USA. 200 µl of 10 µM of either core histones or linker histone (in cell) was titrated against aliquots of 300 µM distamycin (in syringe). Titrations were performed at 25°C under constant stirring at 300 rpm. The resulting thermograms were analyzed using Levenberg – Marquardt non-linear least squares curve fitting algorithm, inbuilt in the MicroCal LLC software. It should be noted that in the low salt buffer used for ITC experiments, the octamer assembly is known to disintegrate [54]. However, due to technical difficulty, it was not possible to perform binding studies in the salt concentrations optimum for octamer integrity.

### Circular Dichroism (CD) spectroscopy

Chromatosome sample (50 µM DNA base), in 5 mM Tris HCl (pH 7.4), 15 mM NaCl was titrated against increasing concentrations of distamycin A solution, in the same buffer. The change in ellipticity, as a function of distamycin concentration was monitored at 25°C using a Spectropolarimeter from BioLogic Science Instruments, France equipped with a Bio-Kine 32 V4.49-1 software. The acquisition duration was fixed at 4 seconds and a wavelength range of 225 to 375 nm was scanned at 0.5 nm intervals. Spectra presented here were obtained by subtraction of buffer baseline, followed by smoothening by moving average method. A similar experiment with chromatosomal DNA served as control.

### In vitro Transcription Assay

*In vitro* transcription of reconstituted chromatin template or an equimolar amount of histone free DNA template was performed in presence and absence of distamycin. The protocol followed, was adapted from Kundu *et al*. 2000 [55] and is detailed in transcription assay figure.

## Results

### Distamycin Affects Chromatosome Stability

**Figure 1:**
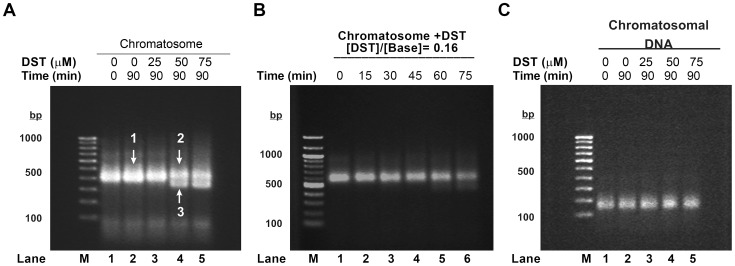
Remodeling of chromatosomes by distamycin A. (A) Agarose gel electrophoresis to study the effect of distamycin on chromatosomes. Chromatosome samples were incubated with distamycin at room temperature for 90 minutes at the drug concentrations indicated and analyzed on 1.5% agarose gel. Chromatosomes incubated with buffer (lanes 1 and 2) served as negative controls. Arrows numbered 1–3 indicate the bands excised and electroeluted for further characterization. (B) Effect of distamycin on chromatosomes, monitored as a function of time. Chromatosome samples (300 µM) were treated with distamycin (50 µM), at room temperature, for varying time intervals, and analyzed on 1.5% agarose gel. (C) Agarose gel electrophoresis to study the effect of distamycin on chromatosomal DNA.l.

In order to study the effect of distamycin on chromatosomes, we have compared the electrophoretic mobility of distamycin treated and untreated rat liver chromatosomes on agarose gel (Figure 1A). Chromatosomes, incubated with distamycin for 90 minutes at room temperature, show a distinctly different pattern of mobility, at and above drug to DNA base ratio of 0.16. There appears a faster migrating population, which is absent in case of chromatosomes, incubated with buffer alone under similar conditions. The smear that appears near about 100 bp corresponds to RNA that has co-purified with chromatosomes. When followed over a course of time, distamycin is observed to affect chromatosomes only after 60 minutes of incubation (Figure 1B). A control experiment with chromatosomal DNA shows no mobility shift (Figure 1C).

For characterization of the species produced upon distamycin treatment, the bands numbered 1–3 in Figure 1A were excised and electroeluted. DNA and histones isolated from the electroeluted samples were then analyzed separately.

**Figure 2:**
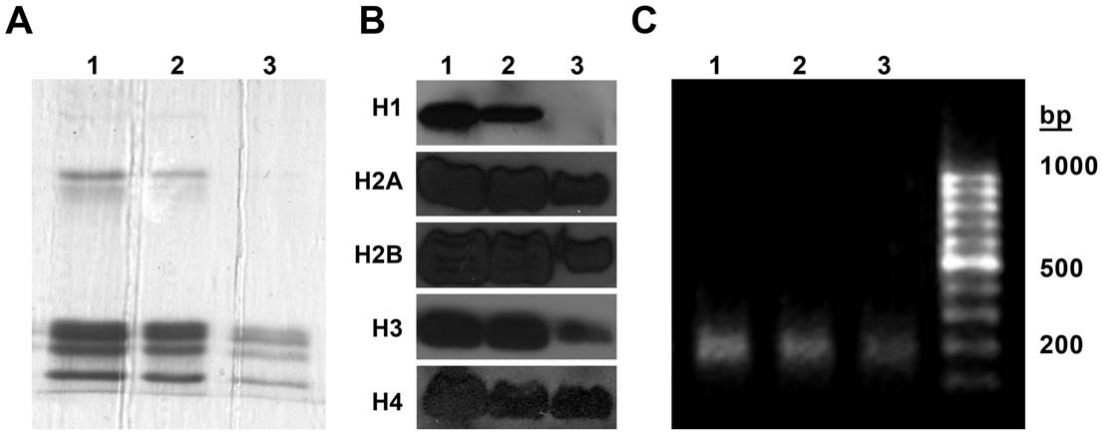
Analysis of remodeled structures. Bands 1–3 of Figure 1A were electroeluted and analyzed separately for their histone and DNA components. (A) SDS-PAGE analysis of histones isolated from the electroeluted samples. Lanes 1–3 contain histones isolated from the corresponding bands in Figure 1A. (B) Western blot analysis of histones present in the SDS-PAGE (Figure 2A). (C) DNA component of the bands 1–3 in Figure 1A. In all the cases, bands 1–3 in Figure 1A correspond to lanes 1–3 in Figure 2.

Figure 2C shows the agarose gel image of the DNA isolated from the electroeluted samples. Lanes 1–3 contain DNA isolated from bands 1–3 of Figure 1A. Co-migration of DNA from all samples indicates that the faster migrating population (band 3 of Figure 1A) is not a distamycin induced DNA cleavage product.

Histones isolated from the electroeluted samples were analyzed on 18% SDS-PAGE (Figure 2A). Lanes 1–3 correspond to histones isolated from bands 1–3 of Figure 1A. It is clearly evident that the faster migrating population (band 3) lacks the linker histone. Western blot analysis of the histone bands (Figure 2B) confirms the same. This indicates that distamycin treatment of chromatosomes leads to eviction of the linker histone.

### Distamycin Induced Remodeling is an ATP Independent Phenomenon

**Figure 3:**
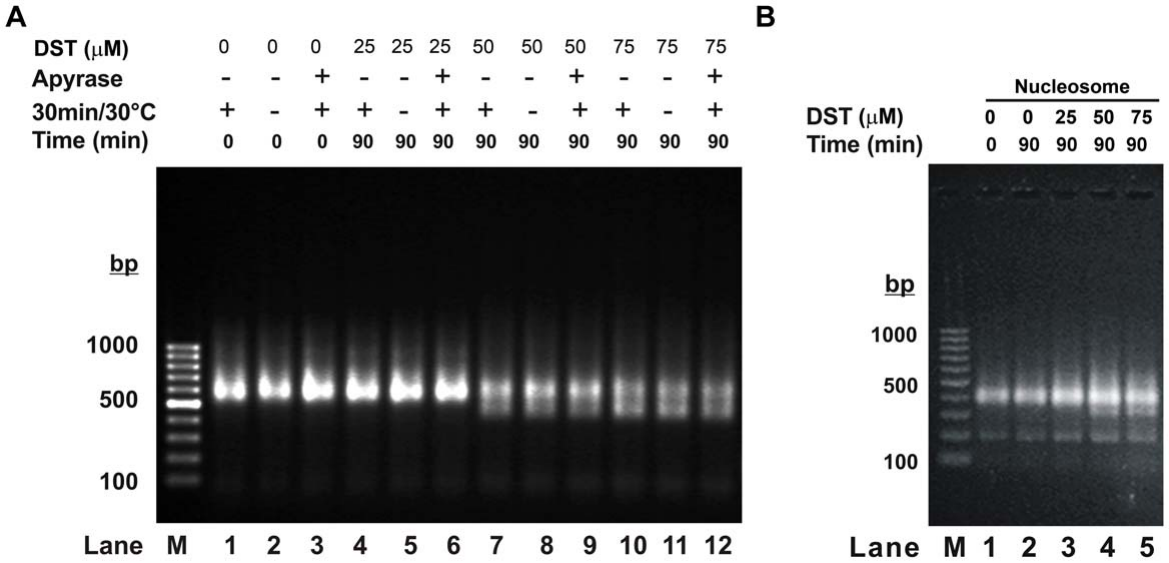
ATP independence of distamycin induced remodeling. (A) Agarose gel electrophoresis to study the effect of distamycin on chromatosomes, with and without prior treatment of apyrase. For apyrase treatment, chromatosomes (300 µM DNA base) were treated with apyrase at 2 U/ml for 30 minutes at 30°C. Chromatosomes were then incubated with distamycin in the drug to DNA base ratios indicated, and electrophoresed on 1.5% agarose gel. (B) Agarose gel electrophoresis to study the effect of distamycin on mononucleosomes, reconstituted on a 200 bp DNA fragment, containing a centrally positioned 601 positioning sequence. Distamycin treatment was performed as indicated.

Distamycin treatment of chromatosomes did not involve the addition of ATP from an external source. However, the chromatosomes, isolated from rat liver, may contain associated ATP that has co-purified in the isolation process. In order to eliminate the contribution of any contaminating ATP in the chromatosome remodeling process, we have repeated the experiments with rat liver chromatosomes, pretreated with apyrase (Figure 3A). Apyrase is a well-established ATP scavenger that has been used to study ATP dependence of remodeling processes in preassembled chromatin templates [53]. Our results indicate that, at and above a distamycin to DNA base ratio of 0.16, apyrase treated chromatosomes also undergo remodeling in a similar manner (Figure 3A, lanes 9 vs 8 and lanes 12 vs 11).

With reconstituted mononucleosomes, the chance of ATP contamination is ruled out. Furthermore, the existence of similar migration pattern on agarose gel (Figure 3B) indicates that in addition to linker histone eviction, distamycin also causes translocation or sliding of the histone octamer. In reconstituted mononucleosomes, the linker histone being absent, the observed effect is perhaps solely due to ‘nucleosomal sliding’. Presence of linker histones (in chromatosomes) generally impedes the restructuring of chromatin [6], [7]. But eviction of linker histone by distamycin seems to render the template competent for remodeling to occur [31]. It may be noted here that a difference in affinity of distamycin for rat liver chromatosomes and nucleosomes, reconstituted on 601 positioning sequence, will not be reflected in the electrophoretic mobility of the species produced.

### Distamycin-histone Interaction Scenario

**Figure 4:**
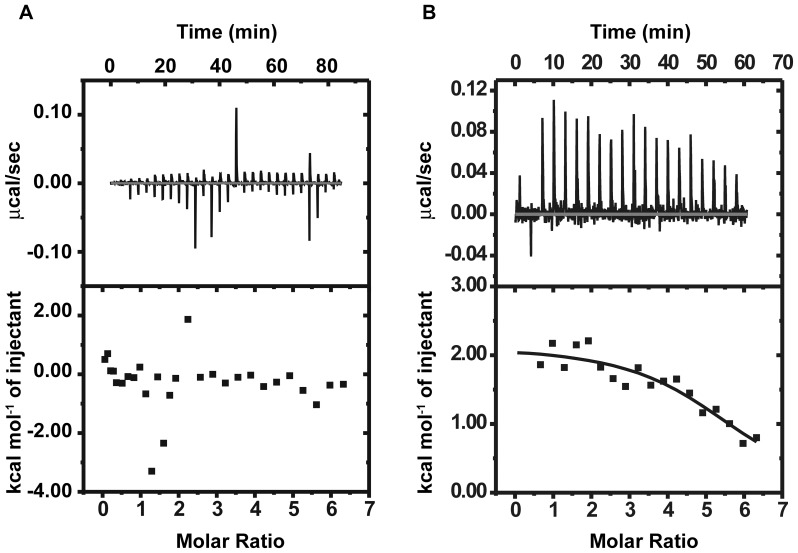
Interaction of distamycin with histones. ITC profiles for the interaction of distamycin with (A) core histones and (B) linker histone in 5 mM Tris HCl (pH 7.4), 100 mM NaCl at 25°C.

ITC experiments of distamycin with core histones show no significant binding, since the ΔH values for the interaction are scattered about 0 Kcal/mol of injectant (Figure 4A). However, there is a modest amount of interaction between distamycin and linker histone (Figure 4B). The least-square fitted parameters (N = 5.72±0.248 Sites, K = 3.36E5±1.81E5 M^−1^, ΔH = 2144±135.0 cal/mol and ΔS = 32.5 calmol^−1^ deg^−1^) indicate an entropy-driven association of the same.

### Characterization of Structural Changes Induced by Distamycin

**Figure 5:**
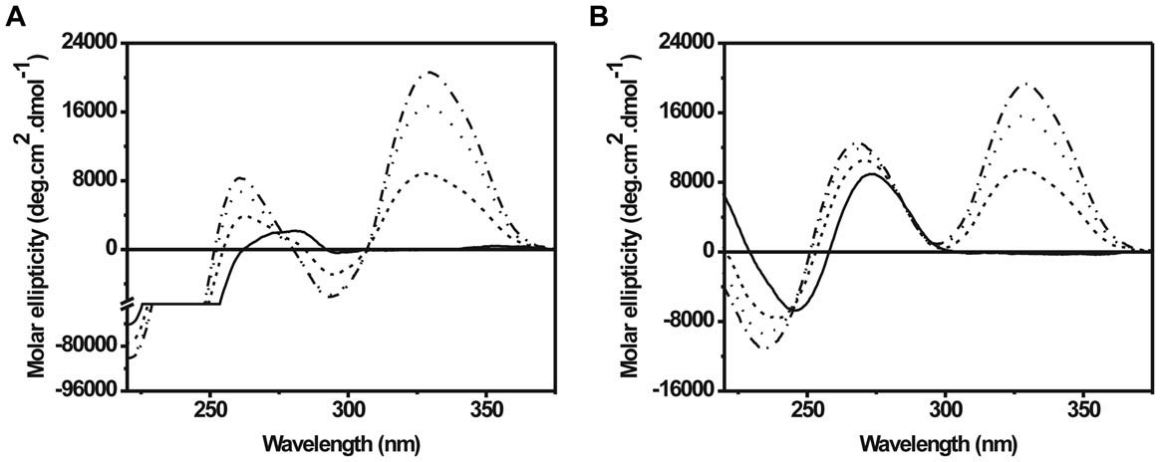
Circular Dichroism spectroscopy to study distamycin induced structural changes of chromatosomes and chromatosomal DNA. (A) Chromatosome or (B) chromatosomal DNA (50 µM nucleotide concentration) is treated with distamycin in drug to DNA base ratios of 0.08 (­­­), 0.16 (·····), and 0.25 (-·-·-).Chromatosome, chromatosomal DNA and distamycin solutions are prepared in 5 mM Tris HCl (pH 7.4), 15 mM NaCl and titrations are performed at 25°C.

We have used circular dichroism (CD) spectroscopy to monitor the structural changes induced by distamycin. Figure 5A shows the CD spectra of chromatosomes in absence and presence of increasing concentrations of distamycin. The CD spectrum of free chromatosomes is intermediate between chromatin and nucleosome core particles. There are two positive maxima around 272 nm and 284 nm that are characteristic of chromatin. However, there is also a small negative signal around 295 nm, which is characteristic of nucleosome core particles [56]. Distamycin addition leads to blue shift of the chromatosome peak. There is also emergence of an induced CD band of bound distamycin with peak around 330 nm. The band intensities increase in a concentration dependent manner. The spectral features of free chromatosome are lost upon addition of distamycin. The peak is gradually shifted to 260 nm. It may be noted here, that chromatosomal DNA peaks around 272 nm (Figure 5B), and a topologically constrained form of plasmid DNA peaks around 260 nm [57]. Our results also show a change in the molar ellipticity below 240 nm, which is generally contributed by histones [58]. This possibly arises due to the interaction of distamycin with linker histones (Figure 4B) since there is no interaction with core histones (Figure 4A). Hence the observed alterations in spectral features may be attributed to the removal of linker histone, and displacement of DNA from the histone core, thereby exposing a constrained DNA stretch.

### Effect of Distamycin on Chromatin Transcription

**Figure 6:**
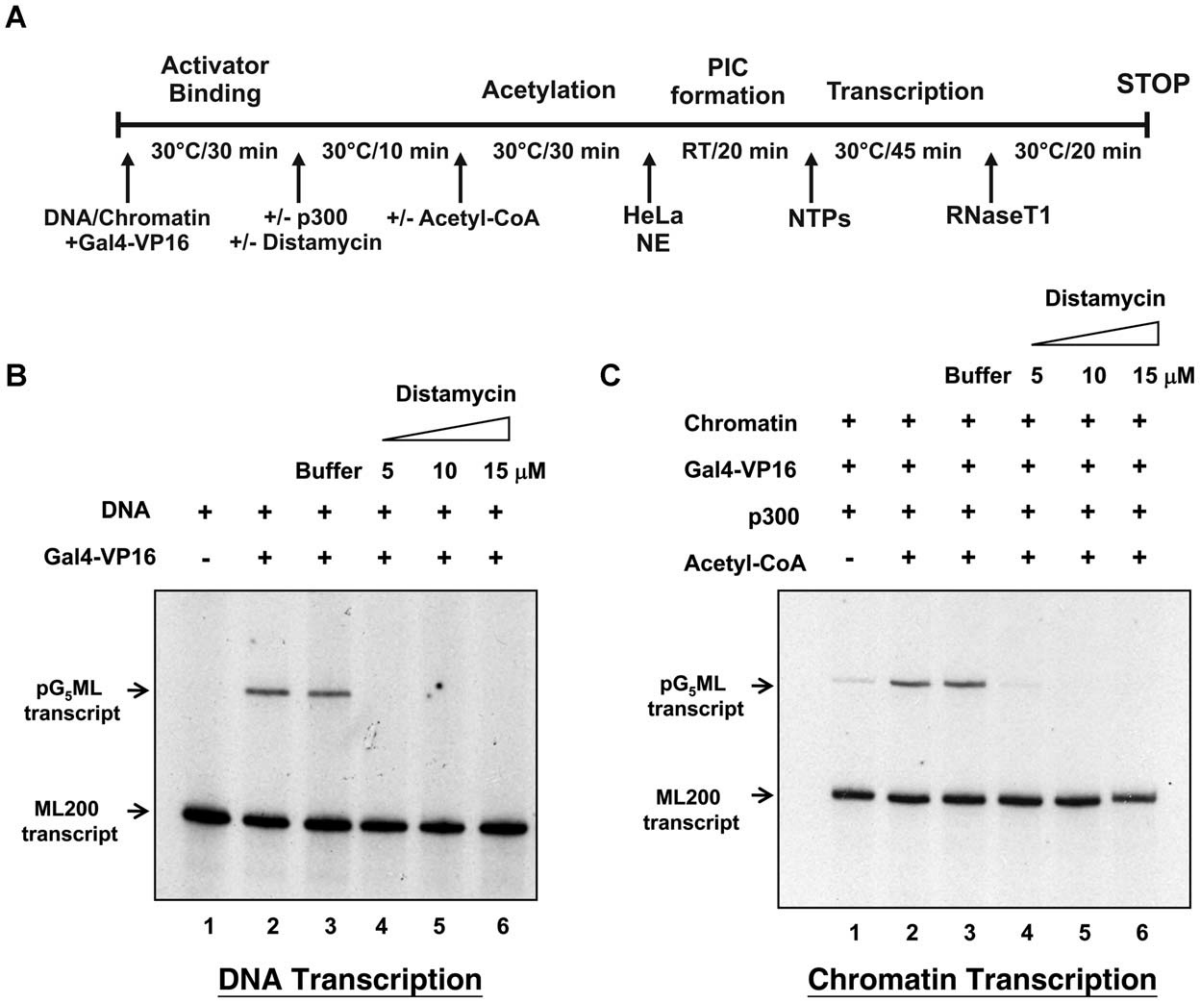
Distamycin inhibits transcription from both DNA and chromatin templates. (A) Schematic representation of the in vitro transcription protocol adopted. Freshly assembled chromatin or an equivalent amount of DNA was subjected to the protocol described in (A). In vitro transcription from DNA template is shown in (B) and p300 Histone acetyl transferase-dependent chromatin transcription is shown in (C). Lane 1 in (B) shows the basal transcription in absence of activator, whereas lane 1 in (C) shows the basal transcription in absence of acetylation (-Ac CoA). Lane 2 in (B) shows activator dependent DNA transcription whereas lane 2 in (C) shows acetylation dependent chromatin transcription. Lane 3 is a buffer control and lanes 4–6 show the transcription profile in presence of increasing concentrations of distamycin.

Enhanced DNA accessibility is generally associated with transcriptional competence. However, earlier studies with DNA template have established the transcription inhibitory potential of distamycin. It was therefore interesting to study the effect of distamycin on transcription from chromatin template. We have performed an *in vitro* transcription assay according to the protocol detailed in Figure 6A [27], [55]. In presence of 5–15 µM of distamycin, there is inhibition of transcription from both naked DNA (Figure 6B, compare lanes 2 and 3 with lanes 4–6) and chromatin (Figure 6C, compare lanes 2 and 3 with lanes 4–6) templates. Chromatin transcription is completely inhibited at and above 10 µM concentration of distamycin.

## Discussion

The accessibility of chromosomal DNA is intimately correlated with its transcriptional competence. Under *in vivo* conditions, the accessibility of DNA is regulated by ATP dependent chromatin remodeling complexes [13] and histone chaperones [59]. To understand the correlation between DNA accessibility and transcription, a relatively new approach involves the use of small DNA binding molecules. In a previous study, Gottesfeld et al. [60] have used a series of minor groove binding pyrrole-imidazole polyamides to investigate any functional relationship between nucleosome mobility and the ability of T7-RNA polymerase to transcribe through chromatin template. Cisplatin and its derivatives have also been used to explore how cisplatin induced cross links affect the structure of nucleosome core particles; whether the adducts inhibit DNA translocation and twist propagation, and how T7 RNA polymerase elongation complexes navigate platinized nucleosomes [61], [62], [63]. However, it should be noted that the gene expression scenario may change markedly in presence of small molecules.

This study focuses on understanding the effect of distamycin on DNA accessibility and transcription. Distamycin A possesses certain interesting properties. It inhibits binding of linker histones to DNA [31], and also changes the rotational positioning of nucleosomal DNA on the octamer surface [50]. We therefore studied its effect on chromatosomes, where the presence of linker histones suppresses the nucleosomal mobility [5], [6], [7]. Similar studies have been performed with reconstituted mononucleosomes, lacking the linker histone.

Our results show that distamycin interacts with chromatosomes forming a species distinctly different from native chromatosomes. The species has higher mobility on agarose gel. Analysis of its DNA and protein component reveals that it lacks the linker histone. However, the DNA component resembles DNA from untreated chromatosomes. Isothermal titration calorimetry indicates an interaction between distamycin and linker histone. Therefore, the linker histones are presumably displaced from chromatosomes as a result of distamycin binding to nucleosomal DNA and the linker histone.

It may be noted that a slight RNA contamination noted in Figure 1A may not contribute significantly to the remodeling reaction since its removal from rat liver chromatosomes (Figure 1B) or absence in reconstituted nucleosomes (Figure 3B) does not alter the results. Similar experiment performed with chromatosomal DNA does not show any change. Apyrase treatment shows that distamycin induced structural changes of chromatosomes occur in absence of ATP.

Remodeling is also apparent in reconstituted mononucleosomes. Similar observations in case of chromatosomes and reconstituted mononucleosomes suggest certain interesting points. In case of chromatosomes, the primary step in remodeling is the eviction of linker histone that in turn renders the template labile. The histone octamer subsequently slides on the DNA. In reconstituted mononucleosomes, the linker histone being absent, the octamer readily translocates on the DNA. However, in order to slide, the octamer has to overcome the energy barrier imposed by its interaction with a high affinity nucleosome positioning sequence. Our current results are insufficient to comment on the formation of any subnucleosomal particles. CD spectroscopy shows that distamycin treatment of chromatosomes gives rise to a structure that contains DNA in topologically stressed form. Since distamycin bends back the helix axis, it is possible that isohelical binding of distamycin to chromatosomal DNA induces torsional stress responsible for the observed effects [64]. The stressed DNA signature hints at the formation of off-centered nucleosomes that exposes a considerable stretch of wrapped nucleosomal DNA. This would be possible if distamycin binding to linker and nucleosomal DNA induces a corkscrew type motion of the DNA with respect to the octamer surface.

Functional consequences of such structural changes were examined by *in vitro* transcription assay. Our results show that distamycin inhibits transcription from both histone-free DNA and chromatin templates. The effect of distamycin upon DNA transcription was shown earlier by Küpper et al., 1973 [40], Puscendorf et al., 1976 [39], and Straney et al., 1987 [65]. They have shown that distamycin inhibits transcription initiation but not elongation. The inhibition takes place by destabilization of the open complex by forcing the promoter to adopt a B-DNA conformation. The structural perturbation is propagated into neighboring DNA. Similar explanations may be applicable in our case as well.

In the context of transcription from chromatin template, there are three major hypotheses to explain transcription inhibition by small molecules [61]: hijacking of transcription factors; physical block for the elongation complex to progress, and inhibition of chromatin remodeling. It is also known that the torsional state of DNA greatly influences promoter unwinding, formation and stability of the open complex, and the escape of RNA polymerase from the promoter. As a result, positive torsional stress induced in a DNA template inhibits transcription initiation, rather than elongation [64], [66], [67].

The results presented here lead us to conjecture that distamycin induced inhibition of DNA and chromatin transcription may arise due to the following reasons: (i) its effect on DNA torsion that in turn affects the twist registry of template DNA; (ii) distamycin may pose a roadblock for the polymerase complex to advance. Present data are insufficient to distinguish between the two possibilities.

This is the first report of a minor groove binder with the potential to induce chromatin remodeling in an ATP-independent manner. However such remodeling reaction is unable to allow transcription. Small molecules with such potential may raise questions on the existing views about gene regulation. They also open up new dimensions that may be explored for further development of cancer therapeutics.
